# Protective and nephrotoxic effects of emodin in animal models of kidney injury: a systematic review and meta-analysis

**DOI:** 10.3389/fphar.2026.1800360

**Published:** 2026-04-02

**Authors:** Guoxiong Liu, Lulu Wu, Ya Li, Xiaohui Chen, Liyuan Yu, Weihang Peng, Li Chen

**Affiliations:** 1 The First Affiliated Hospital of Guizhou University of Chinese Medicine, Guiyang, China; 2 The Second Clinical College of Guangzhou University of Chinese Medicine, Guangzhou, China

**Keywords:** emodin, kidney injury, nephrotoxicity, preclinical evidence, renoprotection

## Abstract

**Background:**

Kidney injury is a growing global health problem with limited treatments. Emodin, an anthraquinone found in traditional Chinese medicinal herbs, shows both renoprotective and nephrotoxic effects in animal studies. This study aimed to systematically assess the effects of emodin on kidney injury *in vivo* and determine how dose, administration route, and treatment duration influence whether the overall outcome is beneficial or harmful.

**Methods:**

A pre-registered systematic review and random-effects meta-analysis was conducted, focusing exclusively on *in vivo* animal models and excluding *in vitro* and clinical data. Eight electronic databases were searched from inception to 1 October 2025. Two reviewers independently performed study selection, data extraction, and risk-of-bias assessment using the SYRCLE tool. The primary efficacy outcomes were serum blood urea nitrogen (BUN) and creatinine (Cr). Pre-specified subgroup analyses were undertaken according to disease model, dose, administration route, and intervention duration. Publication bias was evaluated using funnel plots and Egger’s regression test.

**Results:**

From 2,915 records, 24 studies (408 animals: 396 rodents, 12 canines) met inclusion criteria (17 therapeutic, 7 toxicity). In kidney injury models, emodin significantly reduced BUN (n = 182; SMD = −2.39; 95% CI −3.54 to −1.25; *P* < 0.0001; *I* ^2^ = 78.5%) and Cr (n = 284; SMD = −3.44; 95% CI −4.83 to −2.05; *P* < 0.0001; *I* ^2^ = 86.3%). Subgroup results indicated robust effects at low–moderate doses (<30 mg/kg and 30–60 mg/kg) and with short-to-medium durations; effects attenuated or became inconsistent at higher doses (>60 mg/kg) and with certain routes. For emodin-alone exposure studies, pooled BUN (n = 92; SMD = 2.11; 95% CI −1.51–5.72) and Cr (n = 112; SMD = 0.65; 95% CI −3.15–4.45) showed no overall significant elevation, but the kidney index decreased significantly (n = 100; SMD = −0.68; 95% CI −1.27 to −0.10; *P* = 0.022). High-dose exposure (>1,000 mg/kg), intraperitoneal administration and treatment >1 month were associated with toxicity signals.

**Conclusion:**

Emodin exhibits dual effects on the kidney, with its net outcome determined primarily by dosage, administration route, and treatment duration. These findings underscore the need for precise dosing and exposure control in the further development of emodin as a renal therapeutic agent.

**Systematic Review Registration:**

https://www.crd.york.ac.uk/PROSPERO/view/CRD420261326554, identifier CRD420261326554.

## Introduction

Kidney injury has emerged as a profound global public health problem, with extensive impact and severe consequences (GBD Chronic Kidney Disease Collaboration, 2020; Francis et al., 2024). Systematic reviews indicate that hundreds of millions of people worldwide are affected by kidney disease, which represents a major driver of mortality and disability, and its healthcare burden is projected to continue rising in the future. Currently, therapeutic options to halt the progressive decline of renal function or reverse renal interstitial fibrosis remain limited (Delrue et al., 2024). Therefore, developing translatable interventions that target inflammatory, oxidative stress, and pro-fibrotic pathways holds urgent clinical and scientific significance (Delrue et al., 2024).

Drug development, including that of natural products, often faces a dilemma: many candidate molecules exert therapeutic effects alongside toxicity, which may be an inherent component of their pharmacological activity or an unintended byproduct (Atanasov et al., 2021). This dual nature of efficacy and toxicity presents a twofold challenge in drug development: preserving and amplifying therapeutic benefits while identifying and limiting harmful organ-specific toxicities to ensure a clinically acceptable safety window (Atanasov et al., 2021; Dong et al., 2016).

Emodin is a major anthraquinone component derived from traditional Chinese medicinal herbs such as *rhubarb* and *Polygonum multiflorum* (Dong et al., 2016). Numerous *ex vivo* and animal studies have reported that emodin alleviates inflammation in various models of kidney injury, including acute ischemia-reperfusion injury and diabetic kidney disease, and inhibits fibrosis, thereby improving renal function and histological injury (Feng et al., 2024; Wang et al., 2007; Wang et al., 2023). Concurrently, evidence regarding the nephrotoxicity of emodin cannot be overlooked (Wang et al., 2008). Animal toxicology and cellular studies have reported emodin-associated pathological alterations in renal tubules, apoptosis of renal tubular epithelial cells, and toxic signals such as mitochondrial and lysosomal injury under conditions of high exposure or specific administration settings, suggesting a potential dose-dependent risk of kidney injury (Wang et al., 2008; National Toxicology Program, 2001; Sougiannis et al., 2021).

Thus, emodin exhibits a dual pharmacological character, embodying both therapeutic efficacy and toxicity (Akkol et al., 2021). However, key questions remain unresolved. There is a critical need to untangle how specific experimental or exposure conditions, such as dosage, route of administration, exposure duration, animal species, comorbidities, concomitant medications, and formulation determine whether emodin acts as a renal protective agent or transitions into a nephrotoxic substance (Dong et al., 2016; Akkol et al., 2021). Existing narrative reviews often focus separately on either its protective or toxic effects, lacking systematic, quantitative integration of data across different experimental designs and exposure conditions, and failing to clarify the boundary conditions that determine the direction of the net effect.

To address this critical gap, we conducted a pre-registered systematic review and meta-analysis aimed at quantitatively synthesizing the dual role of emodin in kidney injury models (Page et al., 2021). This study systematically evaluated the efficacy of emodin on major renal function endpoints, such as serum creatinine and blood urea nitrogen, and assessed its nephrotoxicity biomarkers. Through comprehensive sensitivity analyses, risk of bias assessment, and pre-specified subgroup analyses based on administration route, dosage, and intervention duration, we sought to elucidate the key determinants that drive the transition of emodin from a therapeutic agent to a toxicant. This research aims to provide a clear, evidence-based decision-making framework for subsequent development.

## Methods

This systematic review and meta-analysis was designed, conducted, and reported in full compliance with the Preferred Reporting Items for Systematic Reviews and Meta-Analyses (PRISMA) statement, thereby ensuring methodological rigor, transparency, and reproducibility (Page et al., 2021). The protocol has been registered in PROSPERO (CRD420251150142).

### Search strategy

Two independent investigators conducted a systematic literature search across eight databases-PubMed, Web of Science, the Cochrane Library, Embase, CNKI, VIP, Wanfang, and CBM-from database inception through 1 October 2025, to identify relevant animal studies. The primary search strategy combined emodin-related keywords, including “emodin”,“Rheum”, and “Frangulic Acid”, with kidney outcome-related terms, such as kidney injury”, “nephrotoxicity”, and “drug-induced kidney disease”. The specific search strategy is detailed in Supplementary Material S1.

### Inclusion and exclusion criteria

#### Inclusion criteria

Studies were included in this systematic review based on the following PICOS principles:

P (Population): Experimental studies utilizing adult animal models of kidney injury established by chemical or physical methods, such as drug-induced or ischemia-reperfusion injury. Studies must include explicit validation of kidney injury, evidenced by significantly elevated baseline serum creatinine or blood urea nitrogen levels compared to a control group.
I (Intervention): Administration of any dose, via any route, or in any formulation of emodin monotherapy to animals in the experimental group. The intervention may be administered either before or after the induction of the model.
C (Comparison): A control group subjected to the same modeling method and basic procedures as the experimental group must be established. This control group should receive an equivalent volume of solvent or vehicle.
O (Outcomes): Primary endpoint: At least one renal function parameter, such as serum creatinine (Cr) or blood urea nitrogen (BUN) levels, must be reported. For toxicity-focused animal studies, nephrotoxic outcomes were operationally defined based on reported indicators of renal injury, including Cr, BUN, kidney index, and renal histopathological damage when available.
S (Study design): Original, peer-reviewed *in vivo* animal experiments. Language is restricted to Chinese and English.

#### Exclusion criteria

Studies meeting any of the following criteria will be excluded:

Ineligible study types: Reviews, commentaries, conference abstracts, editorials, book chapters, or non-peer-reviewed preprints. *Ex vivo* cell studies, *in silico* (computer simulation) studies, or clinical case reports. Studies with incomplete data, where the full text is unavailable, or where key data cannot be obtained from the authors upon request.

Non-pure intervention:Studies utilizing compound preparations containing emodin and other active ingredients, where the independent effect of emodin cannot be isolated.

Inappropriate control:Studies lacking a suitable control group or employing an unreasonably designed control group, precluding valid comparisons.

Missing outcomes:Studies that do not report the relevant outcomes of interest for this review.

Duplicate publication:For multiple publications from the same research team that potentially use identical animal data, the publication with the most comprehensive data or the highest quality will be included to avoid double-counting.

### Data extraction

Data extraction was independently performed by two investigators following a predetermined standardized protocol. A pre-designed data extraction form was used to systematically collect the following information: basic study characteristics, animal model features, animal sex, emodin intervention details (including dosage, route, and duration), as well as mean renal function values, standard deviations, and sample sizes. All extracted results were cross-checked, and any discrepancies were resolved through discussion or third-party arbitration. Data from figures and tables were obtained by contacting the original authors.

### Risk of bias assessment

The risk of bias assessment for the included studies in this systematic review was independently conducted by two researchers. Any disagreements during the process were resolved through discussion or arbitration by a third researcher. The SYRCLE risk of bias tool for animal studies was utilized for evaluation. This tool systematically assesses potential bias across 10 key domains: (GBD Chronic Kidney Disease Collaboration, 2020): Whether the allocation sequence was adequately generated and applied; (Francis et al., 2024); Whether the baseline characteristics were comparable across groups; (Delrue et al., 2024); Allocation to hide whether sufficient; (Atanasov et al., 2021); Whether the animals were randomly placed during the experiment; (Dong et al., 2016); Whether to blind researchers; (Feng et al., 2024); Whether the animals were randomly selected in the result evaluation; (Wang et al., 2007); Whether the result evaluator adopts the blind method; (Wang et al., 2023); Incomplete data are reported; (Wang et al., 2008); Whether the study report is independent of the report of selective results; (National Toxicology Program, 2001); Whether there is no other bias. Each item was ultimately judged as “yes” (low risk of bias), “no” (high risk of bias), or “unclear” (insufficient information to permit judgment) according to the evaluation criteria.

### Sensitivity and subgroup analysis

To evaluate the robustness of the synthesized results, a sensitivity analysis was performed using the leave-one-out method, in which each study was sequentially excluded and the meta-analysis was repeated to identify any individual study that exerted an excessive influence on the overall effect size. To explore potential sources of heterogeneity among the studies, predefined subgroup analyses were conducted. These analyses were stratified according to key methodological and experimental design variables: animal model type, route of administration, intervention duration, and emodin dose level.

### Assessment and adjustment for publication bias

For outcomes involving more than 10 studies, publication bias was evaluated through visual inspection of funnel plot symmetry, supplemented by Egger’s linear regression test. Publication bias was considered statistically significant when Egger’s test showed *P* < 0.05.

### Statistical analysis

Statistical analyses were performed using R Studio software. For continuous outcomes, effect sizes were calculated as standardized mean differences (SMD) with 95% confidence intervals (CI). A random-effects model was used for all meta-analyses because meaningful between-study heterogeneity was expected across the included preclinical studies with respect to animal species and strains, kidney injury model types, emodin dosing regimens and formulations, administration routes, and treatment duration. A statistically significant difference between the intervention and control groups was considered when *P* < 0.05. The degree of heterogeneity among the results was quantified using the *I* ^2^ statistic. If *I* ^2^ > 50%, sensitivity or subgroup analyses were performed to sequentially examine the sources of heterogeneity and derive more robust conclusions. Publication bias was evaluated by visual inspection of funnel plots and further assessed using Egger’s linear regression test when more than ten studies were included.

## Results

### Study inclusion

**FIGURE 1:**
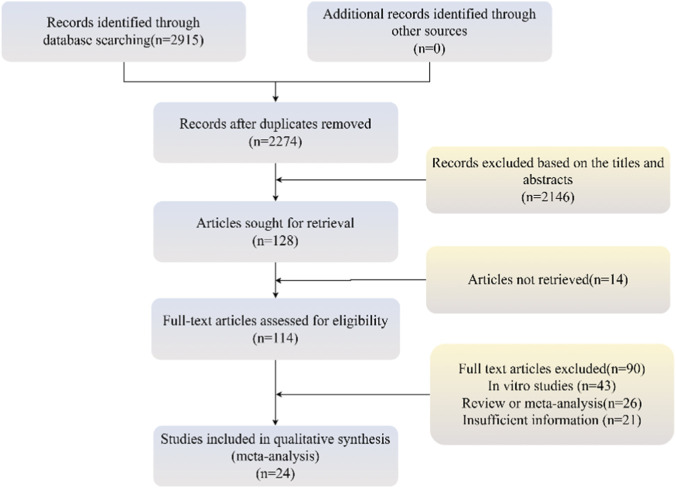
The flowchart of the inclusion process for the studies.

In this study, a total of 2,915 relevant articles were initially retrieved from eight databases, with the specific sources and numbers as follows: PubMed (n = 668), Embase (n = 547), CBM (n = 721), Web of Science (n = 577), CNKI (n = 150), Wanfang (n = 163), VIP (n = 87), and Cochrane Library (n = 2). Ultimately, 24 studies were included for in-depth analysis, among which 17 investigated the therapeutic effects of emodin on kidney injury (Wang et al., 2023; Ali et al., 2011; Cui et al., 2024; Ding et al., 2023; Jia, 2023; Li et al., 2015; Liu, 2017; Lu et al., 2020; Lu et al., 2023; Ou et al., 2023; Qiao et al., 2014; Son et al., 2013; Sun et al., 2019; Wang et al., 2000; Wang et al., 2012; Wang et al., 2022; Yuan et al., 2020), and seven focused on the role of emodin in inducing kidney injury (Huang et al., 2019; Huang, 2019; Huo et al., 2021; Lei et al., 2015; Song et al., 2018; Wang, 2023; Xing et al., 2023). Figure 1 illustrates the complete literature screening process.

### Characteristics of included investigations

Table 1 shows the basic characteristics of the 24 literature. This study involved a total of 408 experimental animals, including 12 canines (4.09% of the total) and 396 rodents. Among the rodents, rats accounted for 62.75% (256/408) and mice for 34.31% (140/408). Based on the research objectives, the included literature was categorized into two groups: one investigating the therapeutic effect of emodin on kidney injury (with kidney injury model groups and emodin intervention groups), and the other examining whether emodin induces kidney injury (with blank control groups and emodin intervention groups). In terms of animal sex, males constituted the vast majority at 87.25% (356/408); three additional studies involved both sexes.

**TABLE 1:** Key characteristics of the 24 included studies.

Study	Species, gender	n = C/E	Model	Dosage of emodin	Treatment of control	Administration method	Duration
Yuan et al. (2020)	BXSB mice, M	6/6	BXSB Model mice	20 mg/kg/d	Saline	i.g	30 d
Ding et al. (2023)	SD rats, M	6/6	Chemical renal injury CLP	60 mg/kg/d	NM	i.p	24 h
Ou et al. (2023)	Wistar rats, M	10/10	Chemical renal injury CLP	35 mg/kg/d	Saline	i.g	28 d
Jia, 2023	Wistar rat, M	6/6	Physical renal injury I/R	40 mg/kg/d	NM	i.p	28 d
Sun et al. (2019)	SD rats, M	6/6	Chemical renal injury Gentamicin Sulfate (i.p.)	50 mg/kg/d	NM	Colonic irrigation	28 d
Wang et al., 2022	C57BL/6 mice, M	8/8	Physical renal injury I/R	10 mg/kg/d	Saline	i.p	12 h
Liu (2017)	SD rats, M	12/12	Chemical renal injury Sodium taurocholate injection	100 mg/kg/d	Saline	i.g	15 d
Li et al. (2015)	SD rats No restrictions	8/8	Physical renal injury Head of pancreas occlusion	100 mg/kg/d	Saline	i.p	7 d
Qiao et al. (2014)	Canine, M	6/6	Physical renal injury I/R	10 mg/kg/d	CMC-Na	i.g	5 d
Wang et al. (2012)	SD rats No restrictions	8/8	Chemical renal injury Sodium taurocholate injection	25 mg/kg/d	Saline	i.p	2 h
Wang et al. (2000)	SD rats, M	15/15	Chemical renal injury STZ (i.p.)	40 mg/kg/d	Saline	i.g	14 d
Ali et al., 2011	Wistar rats, M	6/6	Chemical renal injury Cisplatin (i.p)	10 mg/kg/d	Saline	i.p	24 h
Lu et al. (2020)	SD rats, M	20/20	Physical renal injury I/R	30 mg/kg/d	Saline	i.p	12 h
Son et al. (2013)	SD rats, M	6/6	Chemical renal injury Cyclosporine subcutaneously injection	20 mg/kg/d	Saline	i.h	48 h
Wang et al. (2023)	C57BL/6 mice, M	10/10	Physical renal injury UUO model	40 mg/kg/d	CMC-Na	i.g	7 d
Lu et al. (2020)	SD rats, M	10/10	Physical renal injury 5/6 nephrectomy	4.6 mg/kg/d	Saline	i.g	9 d
Cui et al. (2024)	SD rats, M	5/5	Urosepsis model	0.6 mg/kg/d	Saline	i.g	7 d
Xing et al. (2023)	BALB/c mice, M	6/6	Emodin	800 mg/kg/d	Saline	i.p	14 d
Wang et al. (2023)	SD rats, M	5/5	Emodin	99.2 mg/kg/d	Saline	i.g	28 d
Huo et al. (2021)	KM mice, M	10/10	Emodin	600 mg/kg/d	CMC-Na	i.g	28 d
Huang et al. (2019)	KM mice, M	10/10	Emodin	1600 mg/kg/d	CMC-Na	i.g	77 d
Huang (2019)	KM mice, M	10/10	Emodin	1600 mg/kg/d	CMC-Na	i.g	75 d
Lei et al. (2015)	SD rats, M	5/5	Emodin	1500 mg/kg/d	NM	i.g	16 d
Song et al. (2018)	ICR mice No restriction	10/10	Emodin	100 mg/kg/d	Saline	i.g	14 d

Abbreviation: I/R, Ischemia-reperfusion model; CMC-Na, Sodium carboxymethyl cellulose; C/E, Control/Emodin; UUO, unilateral ureteral obstruction; CLP, cecum ligation and puncture; STZ, streptozozotocin; i.g., intragastric administration; i.p., intraperitoneal injection; i.h., subcutaneous injection; NM, no mention.

In studies focusing on emodin treatment for kidney injury, the animal models used primarily included the following types: nine studies employed chemical injury models induced by intraperitoneal injection of cisplatin, gentamicin sulfate, sodium taurocholate, or uridine, as well as urosepsis and cecal ligation and puncture (CLP) models; seven studies utilized physical injury models, including ischemia-reperfusion, 5/6 nephrectomy, and unilateral ureteral obstruction; the remaining study used the BXSB mouse model, a model for systemic lupus erythematosus.

Regarding emodin administration dosage, in studies on the treatment of kidney injury, eight studies used a dose of 30 mg/kg, seven studies used doses ranging from 30–60 mg/kg, and two studies used doses exceeding 60 mg/kg. In studies on emodin-induced kidney injury, four studies used doses below 1,000 mg/kg, and three studies used doses above 1,000 mg/kg.

In terms of intervention duration, among therapeutic studies, six studies had intervention periods shorter than 2 days, five studies ranged from 2–10 days, and six studies ranged from 10–30 days; among induction studies, four studies had intervention periods shorter than 1 month, and three studies exceeded 1 month.

Regarding the route of administration, in therapeutic studies, intragastric administration (i.g., eight studies) and intraperitoneal injection (i.p., 7 studies) were most commonly used, with one study employing colonic irrigation and one using subcutaneous injection (i.h.). In induction studies, six studies used intragastric administration (i.g.), and one study used i.p.

### Quality evaluation

**FIGURE 2:**
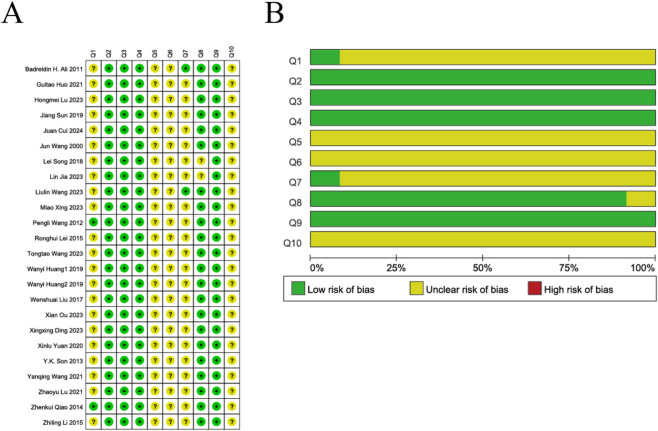
Risk of bias and quality of included studies. **(A)** Risk of bias summary. **(B)** Risk of bias graph.

Overall, the risk of bias assessment indicated that “unclear” ratings were predominant. The included studies generally reported adequate random sequence generation and baseline comparability, with all studies stating that animals were randomly assigned; however, only two studies provided detailed descriptions of the specific randomization methods used. Regarding blinding, only two studies explicitly reported that outcome assessors were blinded, while the remaining studies did not mention any blinding measures. The details for each item in the included literature can be found in Figure 2.

### Emodin in treating kidney injury

#### Outcomes

##### Effect of emodin on pathological injury

Pathological analysis plays a critical role in assessing the degree of kidney injury and validating the therapeutic efficacy of emodin. This meta-analysis revealed that 94% (16/17) of the included studies reported renal pathological data, and all studies utilized hematoxylin and eosin (H&E) staining. Pathological examination demonstrated that the severity of kidney injury in the emodin-treated group was significantly reduced compared with that observed in the model group. Specific improvements included the following: emodin intervention markedly ameliorated renal tissue architecture, alleviated tubular dilatation, and reduced necrosis and exfoliation of renal tubular epithelial cells. Furthermore, it effectively mitigated congestion and edema in the renal interstitium and glomeruli, and significantly decreased inflammatory cell infiltration and nuclear shedding.

##### Effects of emodin on BUN

**FIGURE 3:**
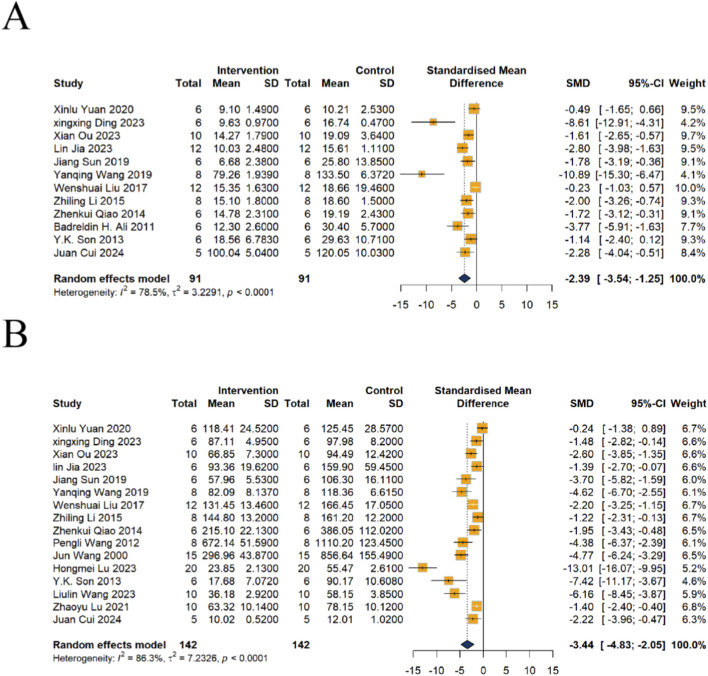
Forest plot of Protective Effects of Emodin on **(A)** BUN and **(B)** Cr in animal models of kidney injury. Data were pooled using a random-effects model. The overall results demonstrated that emodin significantly reduced both BUN (SMD = −2.39, 95% CI: −3.54 to −1.25) and Cr (SMD = −3.44, 95% CI: −4.83 to −2.05).

A total of 12 studies reported changes in BUN. The results demonstrated that emodin significantly reduced the concentration of BUN in kidney injury models, with a pooled n = 182, SMD of −2.39, (95% CI: −3.54 to −1.25, *P* < 0.0001), indicating a statistically significant difference. However, substantial heterogeneity was observed among the included studies (*I* ^2^ = 78.5%, *P* < 0.0001) (Figure 3A).

##### Effects of emodin on Cr

A total of 16 studies reported changes in Cr. The results demonstrated that emodin significantly reduced serum Cr, with n = 284, SMD = −3.44, 95% CI (−4.83, −2.05), *P* < 0.0001, indicating a statistically significant difference. Similarly, substantial heterogeneity was observed among the studies (*I* ^2^ = 86.3%, *P* < 0.0001) (Figure 3B).

#### Subgroup analysis

To investigate potential sources of heterogeneity in this study, subgroup analyses were conducted to explore the effects of animal model types, emodin administration routes, treatment duration, and dosage. Dose-based subgroup thresholds were not pre-specified in the protocol and were selected *post hoc* based on the dose distribution of the included studies, with the aim of facilitating clinically and toxicologically meaningful comparisons. Therefore, dose subgroup analyses were considered exploratory.

##### Subgroup analysis of BUN

**FIGURE 4:**
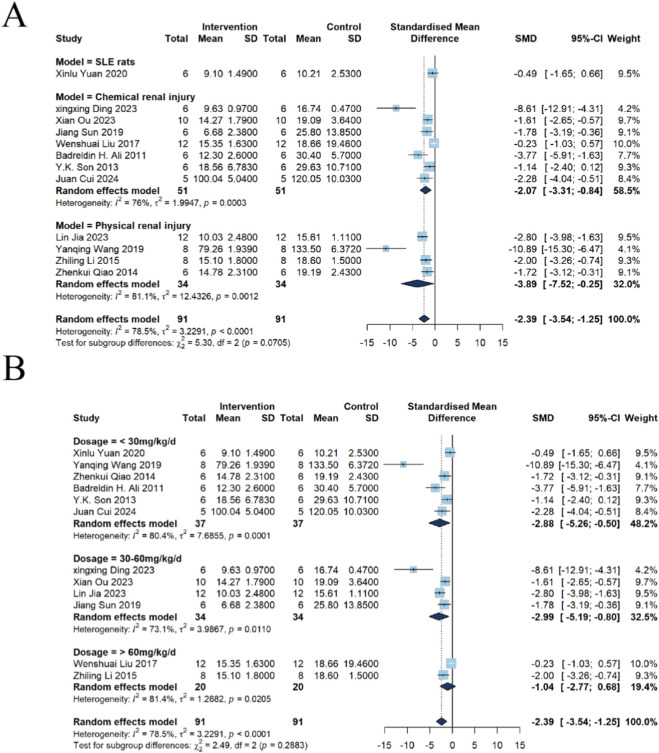
Subgroup analysis of emodin on BUN in kidney injury using a random-effects model. **(A)** Stratified by disease models: significant reductions were observed in chemical (SMD = −2.07, 95% CI: −3.31 to −0.84) and physical (SMD = −3.89, 95% CI: −7.52 to −0.25) injury models, but not in the BXSB autoimmune model. **(B)** Stratified by dosage: significant efficacy was confirmed at <30 mg/kg (SMD = −2.88, 95% CI: −5.26 to −0.50) and 30–60 mg/kg (SMD = −2.99, 95% CI: −5.19 to −0.80), whereas >60 mg/kg showed no significant effect.

Analysis of different model subgroups revealed that among the 12 included studies, 7 employed chemical injury models, 4 utilized physical injury models, and 1 used BXSB autoimmune model mice. Subgroup analysis demonstrated that emodin significantly reduced BUN levels in chemical kidney injury models [n = 102, SMD = −2.07, 95% CI (−3.31, −0.84), *P* = 0.0010; *I* ^2^ = 76%, *P* = 0.0003]. Similarly, a significant reduction in BUN was also observed in physical kidney injury models [n = 68, SMD = −3.89, 95% CI (−7.52, −0.25), *P* = 0.0362; *I* ^2^ = 81.1%, *P* = 0.0012]. However, in the BXSB autoimmune model, no significant effect of emodin on BUN levels was observed [n = 12, SMD = −0.49, 95% CI (−1.65, 0.66), *P* = 0.4030] (Figure 4A).

Analysis of different emodin dosage subgroups revealed the following: among the 12 included studies, six utilized an emodin dosage of <30 mg/kg, four employed 30–60 mg/kg, and two used >60 mg/kg. Subgroup analysis indicated that emodin at <30 mg/kg significantly reduced BUN levels in kidney injury models [n = 74, SMD = −2.88, 95% CI (−5.26, −0.50), *P* = 0.0175; *I* ^2^ = 80.4%, *P* = 0.0001]. A similar reduction was observed in the 30–60 mg/kg dosage group [n = 68, SMD = −2.99, 95% CI (−5.19, −0.80), *P* = 0.0074; *I* ^2^ = 73.1%, *P* = 0.0110]. In contrast, no significant reduction in BUN was detected in the >60 mg/kg dosage group [n = 40, SMD = −1.04, 95% CI (−2.77, 0.68), *P* = 0.2351; *I* ^2^ = 81.4%, *P* = 0.0205] (Figure 4B).

**FIGURE 5:**
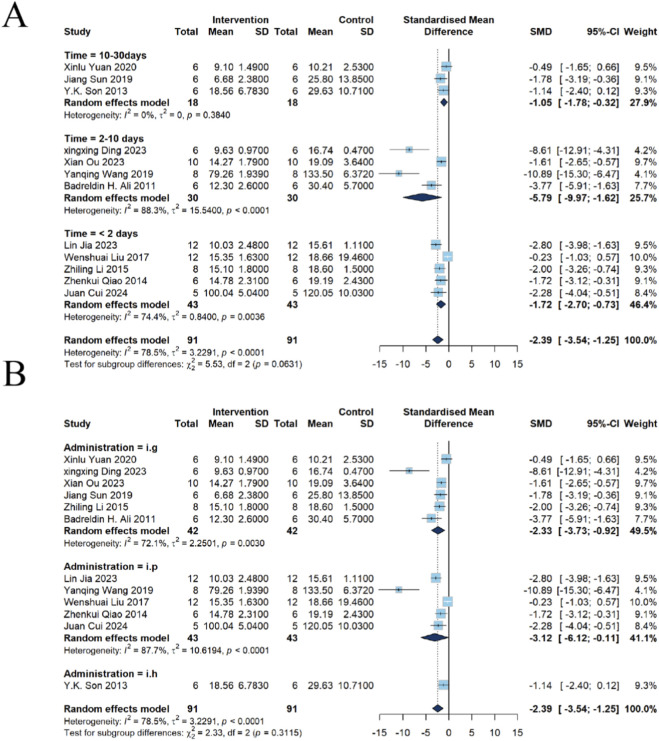
Subgroup analysis of emodin on BUN in kidney injury using a random-effects model. **(A)** Stratified by intervention duration: significant effects were found for <2 days (SMD = −1.72, 95% CI: −2.70 to −0.73), 2–10 days (SMD = −5.79, 95% CI: −9.97 to −1.62), and 10–30 days (SMD = −1.05, 95% CI: −1.78 to −0.32). **(B)** Stratified by administration route: intragastric (SMD = −2.33, 95% CI: −3.73 to −0.92) and intraperitoneal (SMD = −3.12, 95% CI: −6.12 to −0.11) routes significantly reduced BUN, unlike subcutaneous injection.

Analysis of the different administration durations of emodin revealed that among the 12 included studies, five had an intervention duration of <2 days, four had durations of 2–10 days, and three had durations of 10–30 days. Subgroup analysis indicated that emodin administered for <2 days significantly reduced BUN levels in kidney injury models [n = 86, SMD = −1.72, 95% CI (−2.70, −0.73), *P* = 0.0006; *I* ^2^ = 74.4%, *P* = 0.0036]. A BUN reduction was also observed with emodin administered for 2–10 days [n = 60, SMD = −5.79, 95% CI (−9.97, −1.62), *P* = 0.0065; *I* ^2^ = 88.3%, *P* < 0.0001]. Similarly, a consistent reduction was noted in the 10–30 days intervention group [n = 36, SMD = −1.05, 95% CI (−1.78, −0.32), *P* = 0.0048; heterogeneity *I* ^2^ = 0%, *P* = 0.3840] (Figure 5A)This suggests that the intervention duration may be an important contributor to heterogeneity in the BUN (*I* ^2^ = 0%).

Subgroup analysis based on the route of emodin administration was performed. Among the 12 included studies, six used i.g., five employed i.p., and one utilized i.h. The results demonstrated that emodin administered via i.g. significantly reduced BUN levels in animals with kidney injury [n = 84, SMD = −2.33, 95% CI (−3.73, −0.92), *P* = 0.0012; heterogeneity *I* ^2^ = 72.1%, *P* = 0.0030]. Similarly, administration via i.p. also led to a significant reduction in BUN levels [n = 86, SMD = −3.12, 95% CI (−6.12, −0.11), *P* = 0.0423; heterogeneity *I* ^2^ = 87.7%, *P* < 0.0001]. In contrast, i.h. did not exert a statistically significant effect on BUN levels [n = 12, SMD = −1.14, 95% CI (−2.40, 0.12), *P* = 0.0758] (Figure 5B).

##### Subgroup analysis of Cr

**FIGURE 6:**
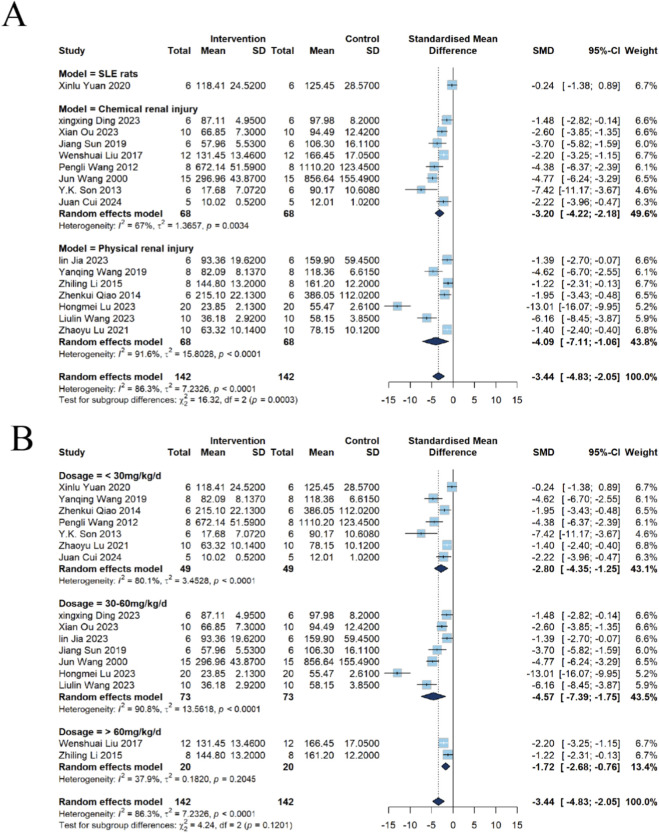
Subgroup analysis of emodin on Cr in kidney injury using a random-effects model. **(A)** Stratified by disease models: significant effects were found for chemical (SMD = −3.20, 95% CI: −4.22 to −2.18) and physical (SMD = −4.09, 95% CI: −7.11 to −1.06) injury models, unlike the SLE rats model. **(B)** Stratified by dosage: <30 mg/kg/d (SMD = −2.80, 95% CI: −4.35 to −1.25), 30–60 mg/kg/d (SMD = −4.57, 95% CI: −7.39 to −1.75), and >60 mg/kg/d (SMD = −1.72, 95% CI: −2.68 to −0.76) dosages significantly reduced Cr.

Among the 16 included studies, eight employed chemical kidney injury models, seven utilized physical kidney injury models, and one adopted the BXSB autoimmune mouse model. Subgroup analysis revealed that in chemical kidney injury models, emodin significantly reduced Cr levels [n = 136, SMD = −3.20, 95% CI (−4.22, −2.18), *P* < 0.0001; *I* ^2^ = 67.0%, *P* = 0.0034]. A significant decrease in Cr was also observed in physical kidney injury models [n = 136, SMD = −4.09, 95% CI (−7.11, −1.06), *P* = 0.0081; *I* ^2^ = 91.6%, *P* < 0.0001]. In contrast, in the BXSB autoimmune model, emodin did not demonstrate statistical significance in altering Cr levels [n = 12, SMD = −0.24, 95% CI (−1.38, 0.89), *P* = 0.6742](Figure 6A).

The results of the subgroup analysis based on different doses of emodin are as follows: Among the 16 included studies, 7 used a dose of <30 mg/kg, 7 used 30–60 mg/kg, and 2 used >60 mg/kg. Subgroup analysis revealed that the <30 mg/kg group significantly reduced Cr levels in the kidney injury model [n = 98, SMD = −2.80, 95% CI (−4.35, −1.25), *P* = 0.0004; *I* ^2^ = 80.1%, *P* < 0.0001]. A reduction was also observed in the 30–60 mg/kg group [n = 146, SMD = −4.57, 95% CI (−7.39, −1.75), *P* = 0.0015; *I* ^2^ = 90.8%, *P* < 0.0001]. A reduction in Cr levels was also noted in the >60 mg/kg group [n = 40, SMD = −1.72, 95% CI (−2.68, −0.76), *P* = 0.0004; *I* ^2^ = 37.9%, *P* = 0.2045] (Figure 6B). This suggests that the dosage may play an important role to heterogeneity in the Cr (*I* ^2^ = 37.9%).

**FIGURE 7:**
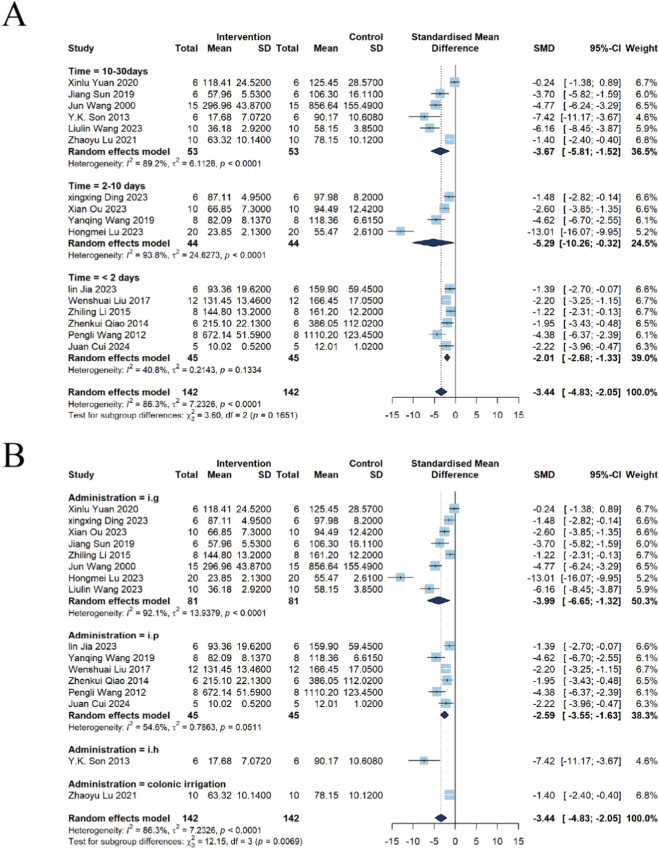
Subgroup analysis of emodin on Cr in kidney injury using a random-effects model. **(A)** Stratified by intervention duration: significant effects were found for <2 days (SMD = −2.01, 95% CI: −2.68 to −1.33), 2–10 days (SMD = −5.29, 95% CI: −10.26 to −0.32), and 10–30 days (SMD = −3.67, 95% CI: −5.81 to −1.52). **(B)** Stratified by administration route: intragastric (i.g) (SMD = −3.99, 95% CI: −6.65 to −1.32), intraperitoneal (i.p) (SMD = −2.59, 95% CI: −3.55 to −1.63), i. h (SMD = −7.42, 95% CI: −11.17 to −3.67), and colonic irrigation (SMD = −1.40, 95% CI: −2.40 to −0.40) routes all significantly reduced Cr.

Subgroup analysis based on the duration of emodin intervention revealed that among all 16 studies, there were 6 studies with intervention durations of <2 days, 4 studies with durations of 2–10 days, and 6 studies with durations of 10–30 days. In the subgroup with an intervention duration of <2 days, emodin significantly reduced Cr levels in kidney injury models [n = 90, SMD = −2.01, 95% CI (−2.68, −1.33), *P* < 0.0001;*I* ^2^ = 40.8%, *P* = 0.1334]. This suggests that the intervention duration may be an important contributor to heterogeneity in the Cr (*I* ^2^ = 40.8%). When the intervention duration was 2–10 days, emodin also demonstrated a Cr-lowering effect [n = 88, SMD = −5.29, 95% CI (−10.26, −0.32), *P* = 0.0369; *I* ^2^ = 93.8%, *P* < 0.0001]. As the intervention duration extended to 10–30 days, this reduction remained consistent [n = 106, SMD = −3.67, 95% CI (−5.81, −1.52), *P* = 0.0008; *I* ^2^ = 89.2%, *P* = 0.0001] (Figure 7A).

Subgroup analysis based on the route of emodin administration revealed that among the 16 included studies, eight utilized i.g. administration, 6 employed intraperitoneal (i.p.) injection, 1 used i.h. injection, and 1 study adopted colonic irrigation. The analysis demonstrated that intragastric administration significantly reduced Cr levels in animal models with kidney injury [n = 162, SMD = −3.99, 95% CI (−6.65, −1.32), *P* = 0.0034; *I* ^2^ = 92.1%, *P* < 0.0001]. Intraperitoneal injection also exhibited a significant Cr-lowering effect [n = 90, SMD = −2.59, 95% CI (−3.55, −1.63), *P* < 0.0001; *I* ^2^ = 54.6%, *P* = 0.0511]. Subcutaneous injection similarly showed a significant reduction in Cr levels [n = 12, SMD = −7.42, 95% CI (−11.17, −3.67), *P* = 0.0001]. Furthermore, colonic irrigation also indicated a decrease in Cr levels [n = 20, SMD = −1.40, 95% CI (−2.40, −0.40), *P* = 0.0060] (Figure 7B).

#### Sensitivity analysis

Sensitivity analysis was performed by systematically excluding each study one at a time. The results demonstrated that, after the removal of any single study, the *I* ^2^ value for the pooled effect did not exhibit a substantial decrease and remained above 50%. This indicates that the high heterogeneity observed in the present study was not driven by any individual study but was widely present across the included set of studies, suggesting the robustness of the findings (Supplementary Material S2, Figures 1, 2).

#### Publication bias

Publication bias was assessed for the primary outcome measures using Egger’s linear regression test. The results indicated visual asymmetry in the funnel plot for BUN, and Egger’s test suggested significant publication bias (t = −6.05, *P* = 0.0001). Similarly, the funnel plot for Cr was also asymmetrical (t = −5.22, *P* = 0.0001). The results suggest a risk of publication bias and should be interpreted comprehensively in combination with subgroup analysis and sensitivity analysis.

### Emodin inducing kidney injury

#### Outcomes

##### BUN

**FIGURE 8:**
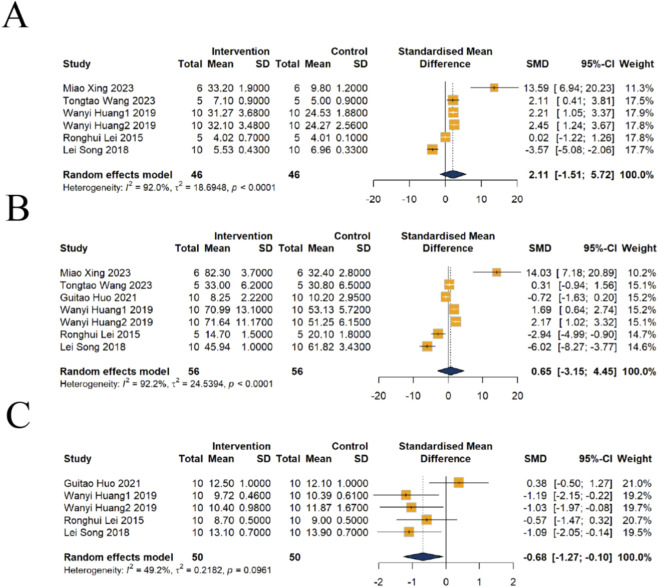
Forest plot assessing the nephrotoxic effects of emodin-alone exposure using a random-effects model. While pooled results for **(A)** overall BUN (SMD = 2.11, 95% CI: 1.51 to 5.72) and **(B)** Cr (SMD = 0.65, 95% CI: -3.15 to 4.45) showed varying trends, a significant decrease was observed in the overall **(C)** kidney index (SMD = -0.68, 95% CI: -1.27 to -0.10), indicating potential nephrotoxicity under certain exposure conditions.

In the analysis assessing the potential nephrotoxicity of emodin, a total of six studies reported BUN. The pooled results indicated no significant elevation in BUN in the emodin group compared with the control group [n = 92, SMD = 2.11, 95% CI (−1.51, 5.72), *P* = 0.2535]; however, substantial heterogeneity was observed among the studies (*I* ^2^ = 92%, *P* < 0.0001) (Figure 8A).

##### Cr

Seven studies reported Cr. The results showed no significant difference in Cr levels between the two groups [n = 112, SMD = 0.65, 95% CI (−3.15, 4.45), *P* = 0.7375], which was also accompanied by high heterogeneity (*I* ^2^ = 92.2%, *P* < 0.0001) (Figure 8B).

##### Kidney index

Furthermore, five studies evaluated the kidney index. The results demonstrated a statistically significant reduction in the kidney index in the emodin group compared with the control group [n = 100, SMD = −0.68, 95% CI (−1.27, −0.10), *P* = 0.0220], with moderate heterogeneity observed (*I* ^2^ = 49.2%, *P* = 0.0961) (Figure 8C).

#### Subgroup analysis

To investigate potential sources of heterogeneity in this study, subgroup analyses were conducted to explore the effects of emodin dosage, administration routes, and intervention duration.

##### Subgroup analysis of BUN

**FIGURE 9:**
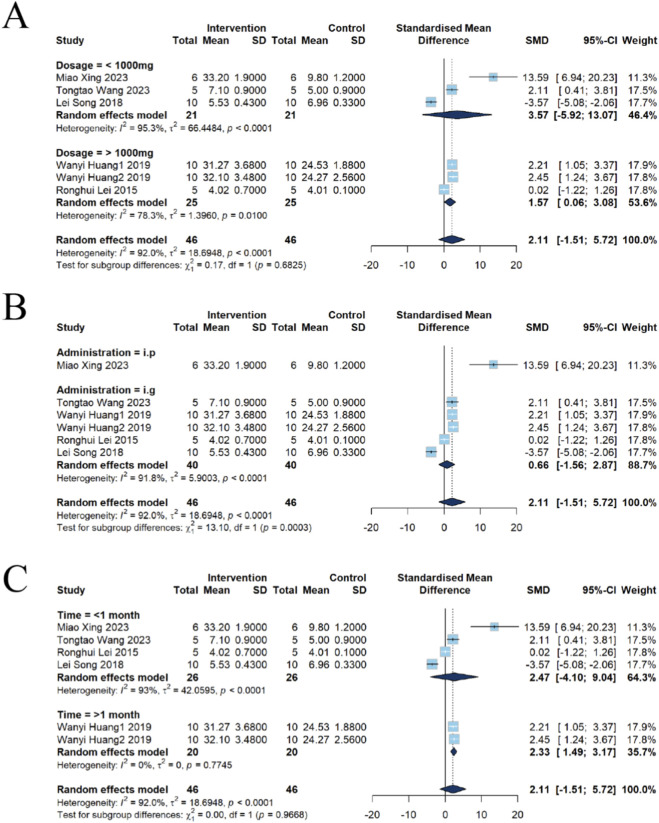
Subgroup analysis of BUN for emodin-induced nephrotoxicity using a random-effects model. **(A)** Stratified by dosage: Significant effects were found for the high-dose group (>1,000 mg; SMD = 1.57, 95% CI: 0.06–3.08), while the low-dose group (≤1,000 mg) showed an SMD of 3.57 (95% CI: −5.92–13.07). **(B)** Stratified by administration route: The intragastric (i.g.) route showed an SMD of 0.66 (95% CI: −1.56–2.87), and the intraperitoneal (i.p.) route showed an SMD of 13.59 (95% CI: 6.94–20.23). **(C)** Stratified by intervention duration: Significant effects were found for >1 month (SMD = 2.33, 95% CI: 1.49–3.17), while <1 month showed an SMD of 2.47 (95% CI: −4.10–9.04).

Dose subgroup analysis indicated that three studies used doses <1,000 mg/kg and three used doses >1,000 mg/kg. Exposure to <1,000 mg/kg of emodin was not associated with a significant change in BUN [n = 42, SMD = 3.57, 95% CI (−5.92, 13.07), *P* = 0.4605; heterogeneity *I* ^2^ = 95.3%, *P* < 0.0001]. By contrast, the >1,000 mg/kg group showed an increase in BUN [n = 50, SMD = 1.57, 95% CI (0.06, 3.08), *P* = 0.0413; heterogeneity *I* ^2^ = 78.3%, *P* = 0.0100] (Figure 9A), suggesting that high-dose exposure may be associated with impaired renal function.

Administration-route subgroup analysis comprised one study employing i.p. and five studies using i.g. Compared with controls, the intraperitoneal group exhibited a marked increase in BUN [n = 12, SMD = 13.59, 95% CI (6.94, 20.23), *P* = 0.0001]. In contrast, oral gavage produced no significant change in BUN [n = 80, SMD = 0.66, 95% CI (−1.56, 2.87), *P* = 0.5618; heterogeneity *I* ^2^ = 91.8%, *P* < 0.0001](Figure 9B). These findings indicate that the route of administration may substantially modulate emodin’s effect on renal function.

Intervention-duration subgroup analysis classified four studies with intervention <1 month and two studies with intervention >1 month. Interventions shorter than 1 month were not associated with significant changes in BUN relative to controls [n = 52, SMD = 2.47, 95% CI (−4.10, 9.04), *P* = 0.4618; heterogeneity *I* ^2^ = 93.0%, *P* < 0.0001]. However, interventions longer than 1 month were associated with increased BUN [n = 40, SMD = 2.33, 95% CI (1.49, 3.17), *P* < 0.0001; heterogeneity *I* ^2^ = 0%, *P* = 0.7745] (Figure 9C), suggesting that prolonged exposure may elevate the risk of nephrotoxicity.

##### Subgroup analysis of Cr

**FIGURE 10:**
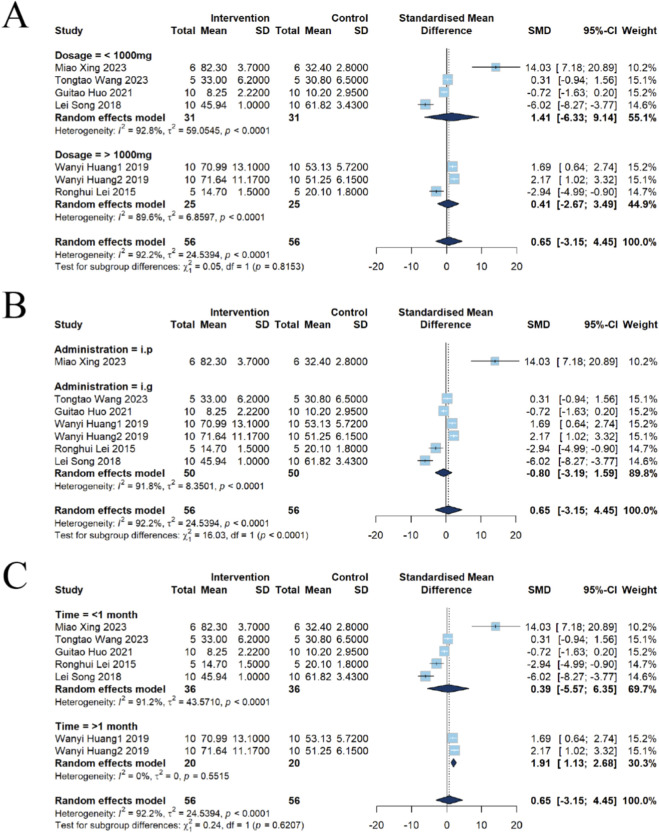
Subgroup analysis of Cr for emodin-induced nephrotoxicity using a random-effects model. **(A)** Stratified by dosage: The low-dose group (≤1,000 mg) showed an SMD of 1.41 (95% CI: −6.33–9.14), while the high-dose group (>1,000 mg) showed an SMD of 0.41 (95% CI: −2.67–3.49). **(B)** Stratified by administration route: The intraperitoneal (i.p.) route showed a significant effect (SMD = 14.03, 95% CI: 7.18–20.89), while the intragastric (i.g.) route resulted in an SMD of −0.80 (95% CI: −3.19 to 1.59). **(C)** Stratified by intervention duration: Significant effects were found for the long-term exposure group (>1 month; SMD = 1.91, 95% CI: 1.13–2.68), while short-term exposure (<1 month) showed an SMD of 0.39 (95% CI: −5.57–6.35).

Dose-response analysis of seven studies revealed that four utilized doses below 1,000 mg/kg, while three employed doses above 1,000 mg/kg. At doses lower than 1,000 mg/kg, no significant change in Cr was observed [n = 62, SMD = 1.41, 95% CI (−6.33, 9.14), *P* = 0.7216], although substantial heterogeneity was present (*I* ^2^ = 92.8%, *P* < 0.0001). Similarly, in the group receiving doses higher than 1,000 mg/kg, no significant increase in Cr was detected [n = 50, SMD = 0.41, 95% CI (−2.67, 3.49), *P* = 0.7926], with high heterogeneity also observed in this analysis (*I* ^2^ = 89.6%, *P* < 0.0001) (Figure 10A).

Analysis by administration route indicated that in one study using i.p., a significant elevation in Cr levels was found [n = 12, SMD = 14.03, 95% CI (7.18, 20.89), *P* = 0.0001]. In contrast, the remaining six studies utilizing i.g. showed no significant change in Cr [n = 100, SMD = −0.80, 95% CI (−3.19, 1.59), *P* = 0.5118], with high heterogeneity noted in this subgroup (*I* ^2^ = 91.8%, *P* < 0.0001) (Figure 10B).

Analysis based on intervention duration suggested that among five studies with an intervention period shorter than 1 month, no significant difference in Cr levels was observed [n = 72, SMD = 0.39, 95% CI (−5.57, 6.35), *P* = 0.8978], and heterogeneity was extremely high *(I* ^2^ = 93.0%, *P* < 0.0001). Conversely, in two studies with intervention durations longer than 1 month, Cr levels were significantly elevated [n = 40, SMD = 1.91, 95% CI (1.13, 2.68), *P* < 0.0001], and heterogeneity in this subgroup was low (*I* ^2^ = 0%, *P* = 0.5515), suggesting that intervention duration may be an important source of heterogeneity among the studies (Figure 10C).

##### Subgroup analysis of kidney index

**FIGURE 11:**
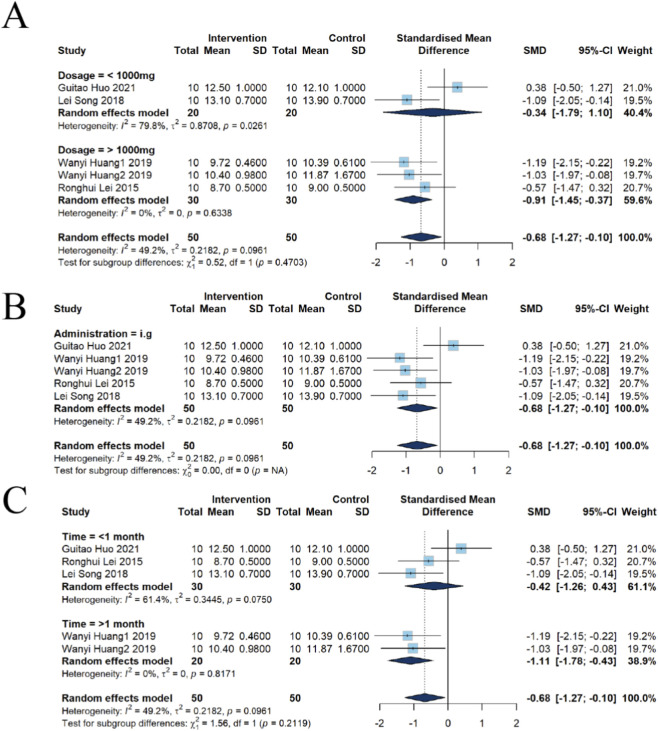
Subgroup analysis of kidney index for emodin-induced nephrotoxicity using a random-effects model. **(A)** Stratified by dosage: A significant decrease was observed in the high-dose group (>1,000 mg; SMD = −0.91, 95% CI: −1.45 to −0.37), while the low-dose group (≤1,000 mg) showed an SMD of −0.34 (95% CI: −1.79 to 1.10). **(B)** Stratified by administration route: The intragastric (i.g.) route significantly reduced the kidney index with an SMD of −0.68 (95% CI: −1.27 to −0.10). **(C)** Stratified by intervention duration: Significant effects were found for long-term exposure (>1 month; SMD = −1.11, 95% CI: −1.78 to −0.43), whereas short-term exposure (<1 month) showed an SMD of −0.42 (95% CI: −1.26 to 0.43).

The results of the dose subgroup analysis are as follows: A total of five studies were included, of which two involved doses <1,000 mg/kg and three involved doses >1,000 mg/kg. Compared with the control group, no significant difference in the kidney index was observed in the <1,000 mg/kg group [n = 40, SMD = −0.34, 95% CI (−1.79, 1.10), *P* = 0.6407; heterogeneity *I* ^2^ = 79.8%, *P* = 0.0261]. In contrast, a significant decrease in the kidney index was observed in the >1,000 mg/kg group [n = 60, SMD = −0.91, 95% CI (−1.45, −0.37), *P* = 0.0009; heterogeneity *I* ^2^ = 0.0%, *P* = 0.6338], with low heterogeneity and relatively consistent and stable results in this subgroup (Figure 11A).

Regarding the route of administration: All five included studies i.g. administration. Overall analysis revealed that intragastric administration significantly reduced the kidney index [n = 100, SMD = −0.68, 95% CI (−1.27, −0.10), *P* = 0.022; heterogeneity *I* ^2^ = 49.2%, *P* = 0.0961], indicating a consistent effect of oral administration on the kidney index (Figure 11B).

The subgroup analysis based on treatment duration indicated: Three studies had an intervention duration of <1 month, and two studies had a duration of >1 month. No significant difference was observed compared with the control group when the intervention duration was <1 month [n = 60, SMD = −0.42, 95% CI (−1.26, 0.43), *P* = 0.3367; heterogeneity *I* ^2^ = 61.4%, *P* = 0.0750]. However, a significant decrease in the kidney index was observed when the intervention duration was >1 month [n = 40, SMD = −1.11, 95% CI (−1.78, −0.43), *P* = 0.0013; heterogeneity *I* ^2^ = 0%, *P* = 0.8171], with low heterogeneity and relatively stable results in this subgroup (Figure 11C).

### Possible mechanism of emodin

To elucidate the dual mechanisms underlying the therapeutic efficacy and toxicity of emodin, this study systematically analyzed its distinct pathways involved in renal protection and toxicity (Table 2).

**TABLE 2:** Possible mechanism of emodin.

Study	Mechanism
Yuan et al. (2020)	Reduces TNF-α, ICAM-1 and FN levels
Ding et al. (2023)	Enhances Nrf2 and AUF1 protein expression, increasing antioxidant capacity
Ou et al. (2023)	Modulates IL-17/NF-κB and TNF/NF-κB signaling pathways
Jia (2023)	Inhibits HMGB1/TLR4/NF-κB inflammatory pathway
Sun et al. (2019)	Regulates gut microbiota balance
Wang et al., 2022	Improves mitochondrial dysfunction
Liu (2017)	Upregulates HIF-1α and GSK-3β
Li et al. (2015)	Upregulates HIF-1α expression
Qiao et al. (2014)	Inhibits oxidative stress and inflammatory factors
Wang et al. (2012)	Reduces TNF-α release
Wang et al. (2000)	Inhibits synthesis and release of ET-1 and TNF-α
Ali et al. (2011)	Enhances activities of antioxidant enzymes (GST, GPx, GR, SOD, CAT)
Lu et al. (2023)	Modulates p53 and HIF-1α/VEGF pathways to inhibit apoptosis
Son et al. (2013)	Inhibits PKCK2 to reduce renal cell apoptosis
Wang et al. (2023)	Inhibits renal fibrosis via the miR-490-3p/HMGA2 pathway
Lu et al. (2020)	Intervenes via microbes and nanomaterials to modulate TLR4/MyD88/NF-κB pathway
Cui et al. (2024)	Activates AMPK/SIRT1 pathway to reduce oxidative stress, inflammation, and complement activation
Xing et al. (2023)	Inhibits the Notch1/Nrf2/GPX4 signaling pathway, inducing ferroptosis
Wang (2023)	Causes disturbances in multiple metabolic pathways, including amino acid, lipid, and energy metabolism
Huo et al. (2021)	NM
Huang (2019)	Induces oxidative stress and inflammatory responses, leading to apoptosis
Huang et al. (2019)	Induces oxidative stress, disrupts mitochondrial function, activates caspase pathways, and modulates MRP1/2 expression, leading to apoptosis and hepatotoxicity/nephrotoxicity
Lei et al. (2015)	NM
Song et al. (2018)	NM

Abbreviation: AUF1, AU-rich element RNA-binding protein 1 (hnRNP D); AMPK, AMP-activated protein kinase; CAT, catalase; ET-1, Endothelin-1; FN, fibronectin; GPx, Glutathione peroxidase (general); GPX4, Glutathione peroxidase 4; GSK-3β, Glycogen synthase kinase-3, beta; GR, glutathione reductase; GST, Glutathione S-transferase; HIF-1α, Hypoxia-inducible factor-1, alpha subunit; HMGA2, High mobility group AT-hook 2; HMGB1, High mobility group box 1; ICAM-1, Intercellular adhesion molecule 1; IL-17, Interleukin 17; MRP1/MRP2, Multidrug resistance-associated protein 1/2; MyD88, Myeloid differentiation primary response 88; NF-κB, Nuclear factor kappa-light-chain-enhancer of activated B cells; Nrf2, Nuclear factor erythroid 2–related factor 2; NM, not mentioned; Notch1, Notch homolog 1 (Notch receptor); p53, Tumor protein p53 (TP53); PKCK2, Protein kinase CK2 (casein kinase-2); SIRT1, Sirtuin 1; SOD, superoxide dismutase; TLR4, Toll-like receptor 4; TNF-α, tumor necrosis factor alpha; VEGF, vascular endothelial growth factor.

#### Mechanisms of renal protection by emodin

##### Anti-inflammatory effects

Studies by Yuan et al. (2020), Wang et al. (2012), and Wang et al. (2000) demonstrated that emodin alleviates inflammatory responses by reducing levels of TNF-α, ICAM-1, and FN, or by inhibiting the synthesis and release of ET-1 and TNF-α. Research by Ou et al. (2023) and Jia (2023) further indicated that its effects are associated with the modulation of the IL-17/NF-κB and TNF/NF-κB pathways, as well as inhibition of the HMGB1/TLR4/NF-κB inflammatory pathway. Lu et al. (2020) proposed that emodin regulates the TLR4/MyD88/NF-κB pathway through interventions involving microbes and nanomaterials.

##### Antioxidant and cytoprotective effects

Studies by Ding et al. (2023) and Ali et al. (2011) revealed that emodin enhances the body’s antioxidant capacity by upregulating Nrf2 and AUF1 protein expression or by increasing the activity of various antioxidant enzymes, such as GST, GPx, GR, SOD, and CAT. Research by Liu (2017) and Li et al. (2015) found that emodin upregulates HIF-1α and GSK-3β, thereby improving cellular tolerance to hypoxia.

##### Anti-fibrotic and anti-apoptotic effects

A study by Wang et al. (2023) showed that emodin inhibits renal fibrosis via the miR-490-3p/HMGA2 pathway. Research by Hongmei Lu (2023) indicated that emodin suppresses apoptosis and promotes angiogenesis by modulating the p53 and HIF-1α/VEGF signaling pathways. Son et al. (2013) discovered that emodin reduces renal cell apoptosis by inhibiting PKCK2.

##### Improvement of cellular function and metabolism

Wang et al. (2022) demonstrated that emodin ameliorates mitochondrial dysfunction. Sun et al. (2019) emphasized its role in regulating the balance of gut microbiota. Juan Cui found that emodin alleviates oxidative stress, inflammation, and complement activation through activation of the AMPK/SIRT1 pathway.

#### Potential mechanisms of emodin toxicity

##### Induction of cell death and injury

A study by Xing et al. (2023) indicated that emodin induces ROS-mediated ferroptosis by inhibiting the Notch1/Nrf2/GPX4 signaling pathway, leading to renal injury.

##### Induction of metabolic disorders and oxidative stress

Research by Wang (2023) revealed that emodin exposure disrupts multiple metabolic pathways, including amino acid, lipid, and energy metabolism. Two studies by Huang et al. (2019) and Huang (2019) confirmed that long-term, high-dose emodin administration induces disturbances in the oxidative system, impairs mitochondrial function, activates caspase-dependent apoptotic pathways, and modulates MRP1/MRP2 expression, ultimately resulting in apoptosis and hepatorenal toxicity.

## Discussion

This systematic review and meta-analysis integrated dual evidence regarding emodin’s therapeutic potential and nephrotoxic risk in kidney disease. The core finding reveals that the biological outcome of emodin-whether protective or toxic-critically depends on key conditions such as dose, route of administration, and intervention duration. As shown in subgroup analyses, this high heterogeneity mainly comes from differences in animal species, injury models, emodin doses, and treatment durations. Regarding data pooling, merging all these diverse studies together can be misleading. Because emodin can be renoprotective at certain doses but nephrotoxic at others, pooling these opposite effects can cause them to mathematically cancel each other out, masking both its true therapeutic benefits and its potential toxicity. Therefore, the overall pooled results should be interpreted with caution. Clinical guidance and translational interpretations should rely heavily on the specific safe doses and conditions identified in subgroup analyses, which we will discuss in detail below.

### Protective

Our primary finding is that emodin intervention demonstrated significant renal function improvement in various kidney damage models, as evidenced by marked reductions in BUN and Cr levels. This aligns with previous reports that emodin exerts renoprotective effects through its anti-inflammatory, antioxidant, and anti-fibrotic properties (Yang et al., 2020; Yu et al., 2022). However, the high heterogeneity observed in the pooled analysis suggests the involvement of more complex factors modulating its efficacy.

#### Chemical and physical versus immune-mediated injury

Subgroup analysis indicated that emodin significantly reduced BUN and Cr in both chemical and physical kidney injury models, suggesting consistent protective effects in non-immune injuries primarily characterized by acute inflammation, oxidative stress, and apoptosis. This is supported by multiple *in vivo* and *in vitro* studies (Wang et al., 2022; Liu et al., 2016):in I/R and drug-induced kidney injury models, emodin alleviated mitochondrial dysfunction, suppressed inflammatory factors and apoptosis, thereby improving renal function parameters. In contrast, the BXSB model, a spontaneous lupus-prone mouse model, features kidney damage primarily driven by immune complex deposition, complement activation, and persistent immune cell infiltration (Almizraq et al., 2022), representing a pathology significantly distinct from acute chemical or ischemic injury. The disease progression and immunological characteristics of the BXSB model may limit detectable improvement in conventional renal function parameters by a single anti-inflammatory or antioxidant agent. Furthermore, the small sample size in the included BXSB group (n = 12) limited statistical power, and the negative results might be partly attributable to insufficient sample size. Therefore, the negative or inconsistent results in the BXSB model could reflect genuine pathological mechanism differences or be constrained by sample size and experimental design. The high heterogeneity (*I* ^2^ > 90%) observed in physical models likely stems from inherent variability in surgical models-factors such as ischemia duration, reperfusion handling, surgical technique, and sampling time significantly impact outcomes (Bromage et al., 2017; Louie et al., 1993). Physical injury models often exhibit high variability across different laboratories, substantially increasing heterogeneity in the pooled effect size.

#### Dose-response and therapeutic window of emodin

Dose subgroup analysis revealed a clear dose-dependent effect of emodin. Within the range of less than 60 mg/kg, emodin significantly improved BUN and Cr levels, with the 30–60 mg/kg dose group showing the strongest effect on serum creatinine reduction, suggesting an optimal therapeutic window. However, at doses exceeding 60 mg/kg, the BUN improvement effect disappeared entirely, and the Cr improvement effect was significantly attenuated, indicating diminished efficacy and inconsistent parameter responses, potentially approaching its toxicity threshold. Concurrently, heterogeneity for Cr results in the high-dose group significantly decreased (*I* ^2^ = 37.9%), indicating that the balanced interplay between the drug’s protective and toxic effects at this stage overshadowed other confounding factors, leading to more consistent results across studies. This finding further supports the risk of emodin transitioning from a protective to a toxic role at high doses.

At low-to-medium doses, emodin primarily alleviates acute kidney injury and reduces BUN and Cr by inhibiting inflammation, activating Nrf2 and downstream antioxidant genes, inhibiting apoptosis, and improving mitochondrial function (Dong et al., 2016). However, when tissue exposure or dosage exceeds a certain threshold, emodin activates/derepresses a series of detrimental pathways, including substantial ROS accumulation, lipid peroxidation, ferroptosis, and even structural and functional damage to mitochondria/lysosomes (Xing et al., 2023), thereby counteracting or reversing its protective effects with toxicity.

#### Short-term rapid benefit, mid-term peak, and long-term stability

Intervention duration subgroup analysis suggested rapid renal protection with short-term administration (less than 2 days). Emodin administration within 2 days post kidney injury resulted in significant decreases in both BUN and Cr. The improvement in serum creatinine was particularly stable (*I* ^2^ = 40.8%), indicating that emodin can rapidly exert protective effects in the acute phase, likely through its potent anti-inflammatory and antioxidant mechanisms. Secondly, medium-term effects (2–10 days) exhibited the strongest efficacy but the least stability. The magnitude of BUN and Cr reduction by emodin peaked during this period, with effect sizes far exceeding those in other periods. However, high heterogeneity was observed among studies, indicating that efficacy during this intervention window is strongly influenced by factors such as inter-individual animal differences and the specific type of injury model. The combination of strong efficacy and instability might suggest that this phase represents both the peak therapeutic period and a balance point between efficacy and potential adverse effects. Furthermore, long-term application (10–30 days) demonstrated effect stability. Although the reduction magnitude for BUN decreased somewhat, the results in the long-term treatment group became highly consistent (*I* ^2^ = 0%). This suggests that after sustained treatment, emodin’s effects enter a stable and predictable plateau phase. This is a positive signal for the long-term management of chronic kidney disease, suggesting its efficacy can be sustained and reliable.

#### Oral stability versus intraperitoneal potency and safety concerns

The impact of different administration routes on emodin’s efficacy and stability requires interpretation considering pharmacokinetics. Overall, i.g. in this meta-analysis significantly reduced both BUN and Cr, thus demonstrating a clear therapeutic signal in animal models; furthermore, oral administration most closely mimics routine clinical dosing and holds high translational value. It is noteworthy that the i.g. subgroup for Cr showed high heterogeneity, likely attributable to variations in intestinal absorption, first-pass metabolism, and inter-animal differences in gut microbiota. These factors significantly influence the final effective exposure reaching the kidneys (Zhou et al., 2023), leading to efficacy fluctuations across studies. Secondly, i.p. demonstrated good efficacy but poor sustainability. It acts very directly, significantly reducing Cr and BUN, and showed relative stability in reducing Cr (*I* ^2^ = 54.6%). This is because injection bypasses the digestive tract, allowing rapid entry into systemic circulation. However, its effect on BUN reduction showed extremely high heterogeneity (*I* ^2^ = 87.7%), and the effect size was not superior to oral administration. Emodin has limited oral bioavailability due to extensive glucuronidation metabolism in the intestine and liver via UDP-glucuronosyltransferase, resulting in lower and more gradual systemic exposure after oral intake (Liu et al., 2012); whereas injection can produce higher transient blood concentrations, more readily approaching the toxicity threshold. Therefore, the safety profile of the same dose may differ drastically depending on the administration route or co-administration with metabolic inhibitors (Di et al., 2015).

In summary, the protective effect of emodin against kidney damage has a defined scope of application. It is effective against kidney injury induced by direct damage such as chemical or physical insults, but may be ineffective against autoimmune nephropathy. Its use requires strict dose control, with 30–60 mg/kg being the optimal range, as exceeding this dose can diminish efficacy. The temporal pattern of its efficacy is distinct: short-term use leads to rapid onset of action, while long-term use results in stabilized effects. Regarding administration route, oral delivery is safer and more reliable than intraperitoneal injection, as the latter, despite faster onset, yields unstable outcomes.

### Nephrotoxicity

Analysis of nephrotoxicity suggests that emodin does carry a risk of renal toxicity; however, this risk manifests only under specific conditions, primarily determined by three key factors: dosage, route of administration, and duration of treatment.

#### Marked nephrotoxicity at doses >1,000 mg/kg

Overall, the results initially appear reassuring-emodin did not significantly increase BUN or Cr levels. Nevertheless, this superficial impression of safety is contradicted by substantial heterogeneity and subsequent subgroup analyses. Crucially, in-depth analysis reveals that emodin-induced nephrotoxicity becomes apparent only under particular circumstances. Dosage is a critical determinant. At doses below 1,000 mg/kg, no significant signs of toxicity were observed in BUN, Cr, or kidney index. In contrast, once the dosage exceeded 1,000 mg/kg, BUN levels increased significantly, and the kidney index decreased markedly. This dose-dependent toxicity profile is highly characteristic, indicating the existence of a relatively safe dosage range.

Recent studies have further confirmed that the dual role of emodin in the kidney is closely related to its systemic exposure. From a pharmacokinetic perspective, emodin exhibits poor oral absorption and significant first-pass effects, leading to highly unstable blood concentrations *in vivo*. This indicates that the effective dose of emodin for renal protection is extremely close to the hazardous dose that induces nephrotoxicity (Feng et al., 2025). Once the administered dose is slightly excessive, resulting in excessively high local drug concentrations in the kidney, emodin can directly induce apoptosis and mitochondrial dysfunction in renal tubular epithelial cells, thereby manifesting significant nephrotoxicity (Liu et al., 2026).

#### Intraperitoneal administration exhibits greatest nephrotoxicity

The route of administration exerts a particularly notable influence. Intraperitoneal injection exhibited the strongest toxicity signals-both BUN and Cr levels increased sharply in this group. By comparison, oral gavage resulted in considerably milder toxic effects. This discrepancy is likely attributable to the fact that intraperitoneal injection bypasses intestinal and hepatic metabolic barriers, allowing a higher concentration of the parent compound to enter systemic circulation and directly damage the kidneys (Liu et al., 2012; Liu et al., 2010).

#### Long-term use induces cumulative renal injury

Duration of treatment represents another crucial variable. Short-term administration for less than 1 month generally did not induce significant abnormalities in renal function parameters. However, long-term use exceeding 1 month was significantly associated with elevated BUN and Cr, as well as a reduced kidney index. This indicates that emodin’s nephrotoxicity exhibits a cumulative effect, with the risk increasing progressively as the treatment duration extends.

#### Kidney index as a sensitive indicator of nephrotoxicity

Notably, changes in the kidney index are particularly informative. Among all parameters, only the kidney index demonstrated a statistically significant difference in the overall analysis. Furthermore, this difference was more pronounced and exhibited low heterogeneity in the high-dose and long-term treatment groups, suggesting robust and consistent findings. This may imply that the kidney index is a more sensitive and reliable indicator for monitoring emodin-induced nephrotoxicity.

Synthesizing these findings, the following important conclusions can be drawn: Emodin does possess nephrotoxic potential, but it manifests only under specific conditions. Safe application requires simultaneous consideration of three boundary conditions: a dosage not exceeding 1,000 mg/kg, preferential selection of the oral route, and controlled treatment duration. The kidney index may serve as a sensitive marker for monitoring emodin-induced renal toxicity. These findings provide clear guidance for the safe use of emodin: by strictly controlling the dosage, selecting an appropriate administration route, and limiting the treatment duration, its nephrotoxic risk can be effectively circumvented, thereby allowing safe utilization of its therapeutic value.

### Mechanisms

Mechanistically, the subgroup analyses in this study can be explained by two interrelated biological pathways. Firstly, regarding dosage, low to medium doses tend to exert protective effects by modulating multiple key molecular pathways. For instance, emodin ameliorates renal inflammation by inhibiting the NF-κB signaling pathway and prevents renal fibrosis through the suppression of the TGF-β/Smad pathway (Feng et al., 2025; Zhang et al., 2025). Concurrently, it activates protective signaling such as Nrf2 and AMPK, thereby rapidly improving BUN and Cr levels through anti-inflammatory effects, enhanced antioxidant defenses, and maintenance of energy homeostasis. Conversely, at excessively high doses, nephrotoxic effects may become more prominent, resulting in paradoxical outcomes. This is potentially due to excessive systemic and renal exposure, altered metabolic handling, and cellular stress responses that exceed adaptive capacity, thereby overwhelming intrinsic protective mechanisms. Specifically, under these stressful conditions, high-dose emodin can disrupt antioxidant axes including Nrf2/GPX4, trigger ROS accumulation and ferroptosis, and paradoxically exacerbate inflammatory or profibrotic cascades, ultimately leading to renal function deterioration and a decreased kidney index. Secondly, intervention duration reveals that short-term treatment primarily confers rapid benefits via anti-inflammatory and antioxidant pathways, whereas long-term or repeated exposure gradually induces structural damage and functional decline due to cumulative oxidative stress, metabolic disorders, and alterations in transporter expression. Overall, the administered dose and exposure duration are pivotal factors determining whether emodin exerts protective effects or induces toxic responses *in vivo*.

Overall, the high heterogeneity (*I* ^2^ > 70%) observed in the primary outcome measures requires careful interpretation. Although subgroup analyses were conducted to explore this heterogeneity, the residual heterogeneity still reflects inherent methodological and biological differences among the included studies, which to some extent limits the generalizability of the pooled results. Specifically, this heterogeneity may stem from the following aspects. Differences among various animal species and strains constitute a non-negligible confounding factor (Zou et al., 2021). The *in vivo* processes of emodin, particularly its significant first-pass effect and dependence on specific intestinal and renal transporters, may differ substantially between mice and rats. Consequently, even with body weight-adjusted dosing regimens, consistent systemic drug exposure levels are difficult to achieve. The synthesis of data from multiple kidney damage models inevitably introduces clinical heterogeneity. For instance, acute kidney injury models, such as ischemia-reperfusion or cisplatin-induced injury, are primarily driven by rapid oxidative stress and renal tubular cell apoptosis (Granata et al., 2022). In contrast, chronic kidney disease models, such as 5/6 nephrectomy or diabetic nephropathy, are characterized by progressive glomerulosclerosis and interstitial fibrosis (Adam et al., 2022). Given the distinct pathological cascades involved, the efficacy of emodin may vary across these models. Therefore, a uniform effect size should not be simply extrapolated to all types of kidney disease. The formulation and vehicle of emodin are also critical variables. Different studies employed various vehicles, such as sodium carboxymethyl cellulose, dimethyl sulfoxide, or normal saline, along with compounds of varying purity. These factors significantly influence the solubility and oral bioavailability of emodin, which itself has extremely poor water solubility (Dong et al., 2016). Variations in intestinal absorption due to formulation differences introduce substantial confounding factors into the data synthesis (Zheng et al., 2021), thereby further limiting the reliability of directly translating these preclinical effect sizes into standardized clinical applications.

### Limitations

Several limitations should be acknowledged when interpreting the present findings. First, substantial heterogeneity was observed in some pooled analyses, which may be related to differences in animal strains, kidney injury models, emodin dosages, administration routes, and treatment durations across studies. Although subgroup and sensitivity analyses were performed to explore potential sources of heterogeneity, residual heterogeneity may still affect the stability of pooled estimates. Second, although dose-based subgroup analyses were used to explore heterogeneity, the dose subgroup thresholds were selected *post hoc* rather than pre-specified. This may introduce data-driven categorization bias; therefore, the dose-stratified findings should be interpreted as exploratory and hypothesis-generating.

Crucially, potential publication bias cannot be entirely ruled out, as suggested by both the asymmetry of the funnel plot and the results of Egger’s test. This inherent bias may lead to an overestimation of the therapeutic efficacy of emodin or an underestimation of its nephrotoxicity risk, as studies with neutral or negative outcomes are often less likely to be published. Finally, caution must be exercised when extrapolating these preclinical findings to clinical practice. Emodin exhibits significant interspecies differences in pharmacokinetics, particularly its extensive first-pass metabolism and renal transport mechanisms. This implies that the specific dose-response relationships and safety thresholds established in rodent models may not be directly applicable to human renal physiology and the complexity of human disease.

Furthermore, frequent omissions in reporting methodological details such as randomization, blinding, and sample size in experimental design may collectively lead to overestimated effect sizes and undermine the reproducibility of research findings. To address these challenges, the field of animal research has progressively established a series of methodological and reporting standards, such as the SYRCLE’s risk of bias tool and the ARRIVE guidelines, aimed at enhancing the rigor of experimental design, the completeness of data reporting, and the reproducibility of results (Hooijmans et al., 2014; Percie et al., 2020)**.**

## Conclusion

This systematic review and meta-analysis confirmed that emodin exerts a typical dual effect on the kidney. At moderate doses and appropriate treatment durations, emodin demonstrates significant renoprotective effects through multiple mechanisms, including anti-inflammatory, antioxidant, and anti-fibrotic activities. However, at excessively high doses or prolonged exposure, it shifts toward inducing oxidative stress and activating cell death pathways, thereby exerting nephrotoxic effects. The transition between these bidirectional actions is strictly dependent on key conditions such as dosage, route of administration, and exposure duration, underscoring the importance of precise dose control and treatment duration management for this natural product. Future primary animal studies should improve methodological reporting transparency, particularly for domains frequently judged as unclear risk in SYRCLE assessments, and should adhere to standardized reporting frameworks such as the ARRIVE guidelines to enhance interpretability and reproducibility.

## Glossary

AMPK: AMP-activated protein kinase
ARRIVE: Animal Research Reporting of *In Vivo* Experiments (guidelines)
AUF1: AU-rich element RNA-binding protein 1 (hnRNP D)
BUN: Blood urea nitrogen
CAT: Catalase
CBM: Chinese Biomedical Literature Database
CI: Confidence interval
CLP: Cecum ligation and puncture
CMC-Na: Sodium carboxymethyl cellulose
CNKI: China National Knowledge Infrastructure
Cr: Creatinine
ET-1: Endothelin-1
FN: Fibronectin
GPx: Glutathione peroxidase (general)
GPX4: Glutathione peroxidase 4
GR: Glutathione reductase
GSK-3β: Glycogen synthase kinase-3 beta
GST: Glutathione S-transferase
H&E: Hematoxylin and eosin
HIF-1α: Hypoxia-inducible factor-1, alpha subunit
HMGA2: High mobility group AT-hook 2
HMGB1: High mobility group box 1
**I** ^**2**^: I-squared statistic (heterogeneity)
ICAM-1: Intercellular adhesion molecule 1
i.g.: Intragastric administration
i.h.: Subcutaneous injection
i.p.: Intraperitoneal injection
IL-17: Interleukin 17
I/R: Ischemia-reperfusion model
MRP1/MRP2: Multidrug resistance-associated protein 1/2
MyD88: Myeloid differentiation primary response 88
NF-κB: Nuclear factor kappa-light-chain-enhancer of activated B cells
NM: Not mentioned/No mention
Notch1: Notch homolog 1 (Notch receptor)
Nrf2: Nuclear factor erythroid 2-related factor 2
p53: Tumor protein p53 (TP53)
PKCK2: Protein kinase CK2 (casein kinase-2)
SIRT1: Sirtuin 1
SMD: Standardized mean difference
SOD: Superoxide dismutase
STZ: Streptozotocin
SYRCLE: Systematic Review Centre for Laboratory animal Experimentation (risk of bias tool)
TLR4: Toll-like receptor 4
TNF-α: Tumor necrosis factor alpha
UUO: Unilateral ureteral obstruction
VEGF: Vascular endothelial growth factor
