# Supplementary material for: Protective and nephrotoxic effects of emodin in animal models of kidney injury: a systematic review and meta-analysis
Source: Front Pharmacol. 2026 Apr 2;17:1800360. doi: 10.3389/fphar.2026.1800360 (PMC13084036; doi:10.3389/fphar.2026.1800360)
Supplement: Supplementary file 1 [file Table1.docx]

**#1**

**(((emodin) OR (Peristim)) OR ((Casanthranol) OR (Frangulic Acid))) OR (((Rheum Emodin) OR (Rheum)) OR ((Frangula Emodin) OR (Archin)))**

**#2**

**Chemically-Induced Kidney Toxicity OR Chemically Induced Kidney Toxicity OR Chemically-Induced OR Kidney Toxicities OR Kidney Toxicities, Chemically-Induced OR Kidney Toxicity, Chemically-Induced OR Toxicities, Chemically-Induced Kidney OR Toxicity, Chemically-Induced Kidney OR Drug-Induced Acute Kidney Injury OR Drug Induced Acute Kidney Injury OR Kidney Injury, Drug-Induced, Acute OR Acute Kidney Injury, Drug-Induced OR Acute Kidney Injury, Drug Induced OR Nephritis, Toxic OR Toxic Nephritis OR Nephrotoxicity, Toxic OR Toxic Nephrotoxicity OR Drug-Induced Kidney Disease OR Disease, Drug-Induced Kidney OR Diseases, Drug-Induced Kidney OR Drug Induced Kidney Disease OR Drug-Induced Kidney Diseases OR Kidney Disease, Drug-Induced OR Kidney Diseases, Drug-Induced OR Drug-Induced Kidney Injury OR Drug Induced Kidney Injury OR Drug-Induced Kidney Injuries OR Injuries, Drug-Induced Kidney OR Injury, Drug-Induced Kidney OR Kidney Injuries, Drug-Induced OR Kidney Injury, Drug-Induced OR Kidney Injury, Drug Induced OR Nephritis, Drug-Induced OR Drug-Induced Nephrotoxicity OR Drug-Induced Nephritis OR Nephrotoxicity, Drug-Induced OR Nephritis, Drug Induced OR Kidney injury**

**#3**

**#1 and #2**
